# Whole Body Awareness for Controlling a Robotic Transfemoral Prosthesis

**DOI:** 10.3389/fnbot.2017.00025

**Published:** 2017-05-30

**Authors:** Andrea Parri, Elena Martini, Joost Geeroms, Louis Flynn, Guido Pasquini, Simona Crea, Raffaele Molino Lova, Dirk Lefeber, Roman Kamnik, Marko Munih, Nicola Vitiello

**Affiliations:** ^1^The BioRobotics Institute, Scuola Superiore Sant'AnnaPisa, Italy; ^2^Robotics and Multibody Mechanics, Flanders Make, Vrije Universiteit BrusselBrussels, Belgium; ^3^Don Carlo Gnocchi FoundationFlorence, Italy; ^4^Laboratory of Robotics at the Faculty of Electrical Engineering, University of LjubljanaLjubljana, Slovenia

**Keywords:** transfemoral amputation, robotic prosthetics, prosthetics control, wearable sensors, sensory fusion, whole body awareness

## Abstract

Restoring locomotion functionality of transfemoral amputees is essential for early rehabilitation treatment and for preserving mobility and independence in daily life. Research in wearable robotics fostered the development of innovative active mechatronic lower-limb prostheses designed with the goal to reduce the cognitive and physical effort of lower-limb amputees in rehabilitation and daily life activities. To ensure benefits to the users, active mechatronic prostheses are expected to be aware of the user intention and properly interact in a closed human-in-the-loop paradigm. In the state of the art various cognitive interfaces have been proposed to online decode the user's intention. Electromyography in combination with mechanical sensing such as inertial or pressure sensors is a widely adopted solution for driving active mechatronic prostheses. In this framework, researchers also explored targeted muscles re-innervation for an objective-oriented surgical amputation promoting wider usability of active prostheses. However, information kept by the neural component of the cognitive interface deteriorates in a prolonged use scenario due to electrodes-related issues, thereby undermining the correct functionality of the active prosthesis. The objective of this work is to present a novel controller for an active transfemoral prosthesis based on whole body awareness relying on a wireless distributed non-invasive sensory apparatus acting as cognitive interface. A finite-state machine controller based on signals monitored from the wearable interface performs subject-independent intention detection of functional tasks such as ground level walking, stair ascent, and sit-to-stand maneuvres and their main sub-phases. Experimental activities carried out with four transfemoral amputees (among them one dysvascular) demonstrated high reliability of the controller capable of providing 100% accuracy rate in treadmill walking even for weak subjects and low walking speeds. The minimum success rate was of 94.8% in performing sit-to-stand tasks. All the participants showed high confidence in using the transfemoral active prosthesis even without training period thanks to intuitiveness of the whole body awareness controller.

## Introduction

Transfemoral amputation is a major disabling condition and early rehabilitation intervention is essential for regaining adequate level of mobility, thus independence in daily life (Pohjolainen et al., 1990). Among lower-limb amputations, proximal amputations such as transfemoral ones, have severe consequences on the future mobility and person's quality of life. A transfemoral amputee—particularly if for dysvascular morbidity—walks more slowly (40% lower than regular gait speed) and consumes 2.5 times more energy than healthy individuals (Waters et al., 1976). Exhaustion due to enormous physical and cognitive effort spent in motor re-learning is the main cause of ineffective treatment. As a consequence of frustration of walking with passive prosthesis and stump-related issues, about 80% of dysvascular transfemoral amputees prefer wheelchairs and accept the associated reduced mobility (Ephraim et al., 2003).

The majority of commercially available devices are passive or semiactive prostheses capable of storing and releasing energy at different phases of the gait cycle (Hafner et al., 2002). Though they are designed to restore a more natural gait pattern during walking, they cannot fulfill power requirements needed to assist the amputee in other locomotion-related activities. In recent years, research in wearable robotics fostered the development of innovative active mechatronic lower-limb prostheses designed with the goal to reduce the cognitive and physical effort of lower-limb amputees in rehabilitation and daily life activities. These devices also enable execution of those tasks requiring active power delivery at the level of the knee joint (for instance, walking over slopes and climbing/descending stairs using reciprocal gait). As a result, devices constituted by active knees, active ankle joints or integrated ankle–knee systems were successfully developed (Windrich et al., 2016) and some of them, e.g., the Power Knee by Ossur (Ossur, Reykjavik, Iceland), are commercially available.

Despite promising perspectives, their adoption is still quite limited by usability-related issues. Beside physical-related aspects such as weight, cosmetics, and noise emissions, the interaction with a device constituted by multiple active joints replacing the natural limb movements demands high cognitive load to the amputee. To ensure benefits to the users, active mechatronic prostheses are expected to be aware of the user intention and properly interact in a closed human-in-the-loop paradigm. Intuitiveness of the controller remains the hardest challenge in the design of powered prostheses.

Early approaches were based on mimicking natural movements by means of echo controllers mirroring the sound limb kinematic on the prosthetics segments (Jimenez-Fabian and Verlinden, 2012; Tucker et al., 2015) or on pattern generators reproducing the natural periodicity of gait and its minor intra-cycle variations for continuous joint actuation (Jimenez-Fabian and Verlinden, 2012; Tucker et al., 2015). Nevertheless, obtrusive and unpredictable movements can be difficultly managed by a system based on gait periodicity. A valid approach for the exploitation of gait periodicity and the flow of subsequent phases during which both the knee and ankle show specific behavior is represented by finite-state machines (Jimenez-Fabian and Verlinden, 2012; Tucker et al., 2015). Reliability of the interface is another critical aspect for the final usability of those control policies translating the human intentions in actuation commands at the level of the powered articulations. In the state of the art, various cognitive interfaces have been proposed to estimate the user's intention online. Effective attempts were based on pattern recognition performed on the measurements from different sensors modalities.

Surface electromyography (sEMG) is a widely adopted solution for directly driving active mechanisms of mechatronic prosthetics relying on information directly linked to the peripheral neuro-muscular system performing pattern recognition based on linear techniques like linear discriminant analysis (LDA; Jin et al., 2006; Huang et al., 2009) or non-linear ones, such as support vector machines (Ceseracciu et al., 2010). Researchers are also exploring targeted muscles re-innervation for an objective-oriented surgical amputation promoting wider usability of active prostheses (Hargrove et al., 2013). However, this procedure cannot be performed in most amputation cases. More successful approaches (still based on LDA) have proposed integrated neuromechanical sensory fusion (Huang et al., 2011; Tkach and Hargrove, 2013; Young et al., 2014) combining sEMG with mechanical sensors embedded in the prosthetic segments such as, pressure sensors, load cells, or inertial measurement units (IMUs). Neuromechanical approaches ensured higher accuracy rates with respect to considering solely sEMG. Nevertheless, the presence of sEMG is often a limit to the robustness in long-term utilization. The quality of the sEMG information is highly affected by sensor's placement and donning, and it is prone to fade in prolonged use to skin perspiration (Abdoli-Eramaki et al., 2012), temperature (Winkel and Jørgensen, 1991), conductance (Nordander et al., 2003). Unstable performance of the sEMG-based interface undermines the robustness of the prosthesis controller. Given the disadvantages of sEMG and consequently of neuromechanical sensory fusion, alternative approaches have led to identify control methods based only on integrated mechanical sensors.

Few approaches fully investigated the exploitation of mechanical sensing as cognitive interface for powered devices including insole pressure sensing (Wang et al., 2013; Chen et al., 2015; Yuan et al., 2015), capacitive sensing (Zheng et al., 2014), load cells (Varol and Goldfarb, 2007), and IMUs (Maqbool et al., 2016) to identify transitions in lower limb prostheses with high accuracy. These methods lacked predictive capabilities as most predictions of terrain occurred after ground contact performing fuzzy-logic event-based (Yuan et al., 2015) or k-Nearest-Neighbor classification or implementing discrete finite state machines (Maqbool et al., 2016).

In a recent work by Spanias et al. (2016), the authors built a classifier that, thanks to linear discriminant analysis, is capable to disregard the EMG signals when corrupted by long-term use and switch to mechanical sensing-based recognition for preserving safety.

Recent advancements have implemented environment awareness controller by means of depth or infrared sensing (Zhang et al., 2011; Krausz et al., 2015; Liu et al., 2016) to detect different terrains; nevertheless, strong reliance on the sensor's line of sight is one of the main limitations of such approach.

The objective of this work is to present an extension of the whole body awareness controller developed for the CYBERLEGs prosthesis preliminarily presented in Ambrozic et al. (2014). The controller, currently implemented on the advanced version of the alpha-prototype—namely the beta-prototype—relies on a distributed non-invasive wearable interface constituted of a set of IMUs and a pair of sensitive instrumented shoes. Non-invasiveness of the sensory apparatus is also reflected by its ease of use. The instrumented shoes can be worn as common sneakers; the IMUs are placed on elastic belts that the user can wear over clothes. By monitoring the subject's movements and kinematics wirelessly online, the whole body awareness controller (WBAC) drives the prosthesis through a double layered control architecture. The high level layer decodes on the basis of a simple set of heuristic transition rules the intention of the user and closes the loop on the human counterpart by commanding the proper setpoints to the low-level controllers of the active knee and ankle joints. Four transfemoral amputees participated to an experimental validation covering activities from treadmill walking, ground-level walking on parallel bars, stairs ascent and sit-to-stand/stand-to-sit maneuvres. All the participants were able to complete the protocol showing high confidence in using the active trasnfemoral prosthesis (ATP) without long training period thanks to intuitiveness of the WBAC.

The rest of the paper is structured as follows: Section Materials and Methods describes the mechatronic system composed by the ATP, the wearable sensory interface and the controller. Then, the experimental protocol and the methods for data analysis are presented. Section Results reports the results of the experimental validation. Section Discussions and Conclusions discusses the results and draws the conclusions.

## Materials and methods

**Figure 1:**
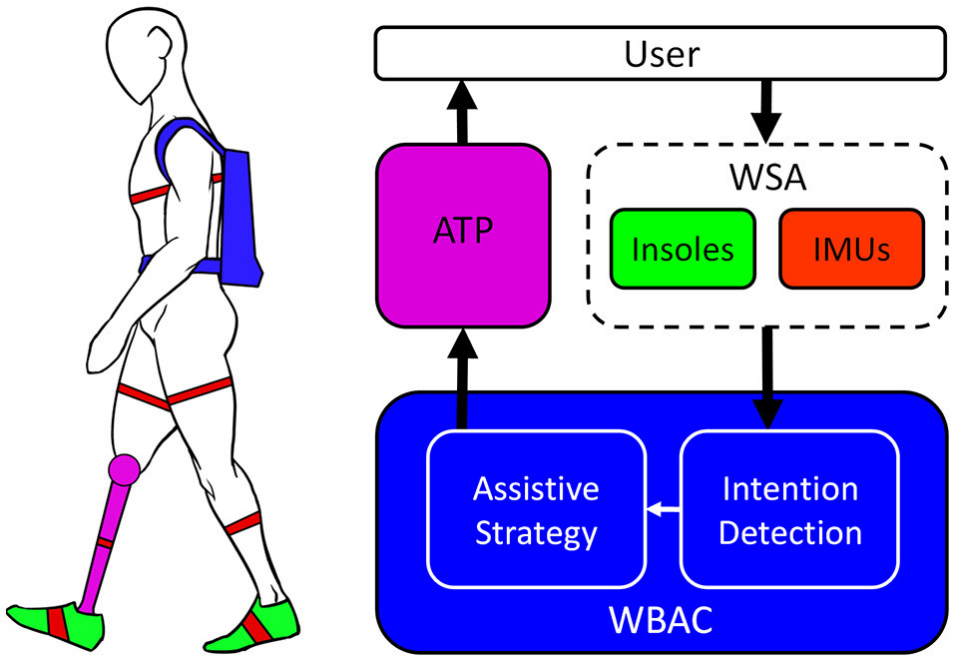
Schematic overview of the main components of the WBAC for the CYBERLEGs ATP. The ATP is controlled by means of WBAC monitoring signals from the WSA composed of 7 IMUs and a pair of instrumented sensitive shoes. Sensory fusion and intent recognition are performed in the real-time controller integrated in the backpack endowed with Zigbee receiver for acquiring signals from the WSA and with batteries for operating the ATP.

The WBAC described in this study is part of an integrated system conceived to ideally achieve effortless control over an ATP by combining a whole body aware cognitive interface with a real-time intention detection algorithm, with the ultimate goal of reducing the effort required for amputees' locomotion. The overall system includes three main parts: (i) an ATP, namely the CYBERLEGs ATP, (ii) a Wearable Sensory Apparatus (WSA) acting as a cognitive interface and (iii) a subject-independent controller merging the information sensed by the WSA to detect subject's intentions and accordingly feed commands to the actuators (Figure 1).

### The CYBERLEGs ATP

The CYBERLEGs ATP was developed by Vrije Universiteit Brussel, starting from the knowledge gained by the engineering of its first version (Geeroms et al., 2013; Flynn et al., 2015a). The design of the current prototype displays minor changes in the ankle joint—still relying on variable compliant actuation while adding parallel compliance—while the knee joint has been substantially modified in order to enable active power delivery, contrarily to the first design as a semi-active joint (Flynn et al., 2015b; Geeroms et al., 2017). Such optimization results in successful replication of the knee torque-angle characteristics thanks to the combined action of three subsystems: the Weight Acceptance, the Energy Transfer, and the Knee Drive. Locking the Weight Acceptance before the heel-strike, that is engaging a high stiffness spring, results in high joint stiffness, producing the required support for the weight acceptance phase, while engagement of the Energy Transfer mechanism during late stance has the two-fold outcome of yielding plantar flexion which complements the ankle actuation action for the push-off and providing the extensor torque necessary to avoid knee collapse; the Knee Drive represents the added key feature: an actuator is mounted in series with two opposing springs, resulting in a Series Elastic Actuation configuration, that allows to actively drive the knee joint (Flynn et al., 2015b; Geeroms et al., 2017).

This new design enables to actively inject energy from the knee, thus ensuring active motion of the prosthesis also during locomotor tasks requiring active movements from the knee, such as stair ascent and sit-to-stand maneuvres, which could not be assisted with the previous passive joint.

### The wearable sensory apparatus

The cognitive interface is implemented as a network of distributed, non-invasive wearable sensors made up of two pressure-sensitive insoles, developed at Scuola Superiore Sant'Anna of Pisa, and a set of seven IMUs, engineered by University of Ljubljana, placed on user's lower-limb segments and trunk. The first exploits an array of optoelectronic pressure transducers placed under the foot to provide information about ground reaction forces and plantar pressure distributions (Crea et al., 2014), while the latter tracks lower-limb segments' kinematics from the orientation of each unit, extrapolated with Kalman filtering and quaternions from raw data measurements (Beravs et al., 2011). WSA communication network is based on the IEEE 802.15.4 wireless protocol. The communication module uses 4 DiZiC DZ-ZB-GX transceiver modules for communication with the IMUs and insoles.

The information sensed by each component is wirelessly transmitted to the central control unit, where real-time merging and processing of all the acquired data returns the input information for the intention detection algorithm.

### The controller

The control of the CYBERLEGs ATP is grounded on the same rationale as the strategy developed for the first prototype, which was presented in Ambrozic et al. (2014): the signals acquired through the WSA are fed to a finite-state machine implementing subject-independent real-time intention detection and consequently driving the actuators. Basically, the state of the user is decoded evaluating subject's lower-limbs kinematics, according to threshold-based transitions rules. Definition of the transition rules is the result of three main types of activities: (i) previous studies conducted with healthy and amputee subjects (Ambrozic et al., 2014; Goršič et al., 2014); (ii) offline analysis of data collected from healthy subjects wearing only the WSA while performing stair ascending and sit-to-stand/stand-to-sit; (iii) offline analysis of data collected from amputee subjects wearing only the WSA while performing stair ascending and sit-to-stand/stand-to-sit. In the novel instance of the WBAC, detection and assistance of supplementary maneuvres are implemented, since the design of the ATP allows for active support during additional tasks requiring active injection or dissipation of energy from the knee.

**Figure 2:**
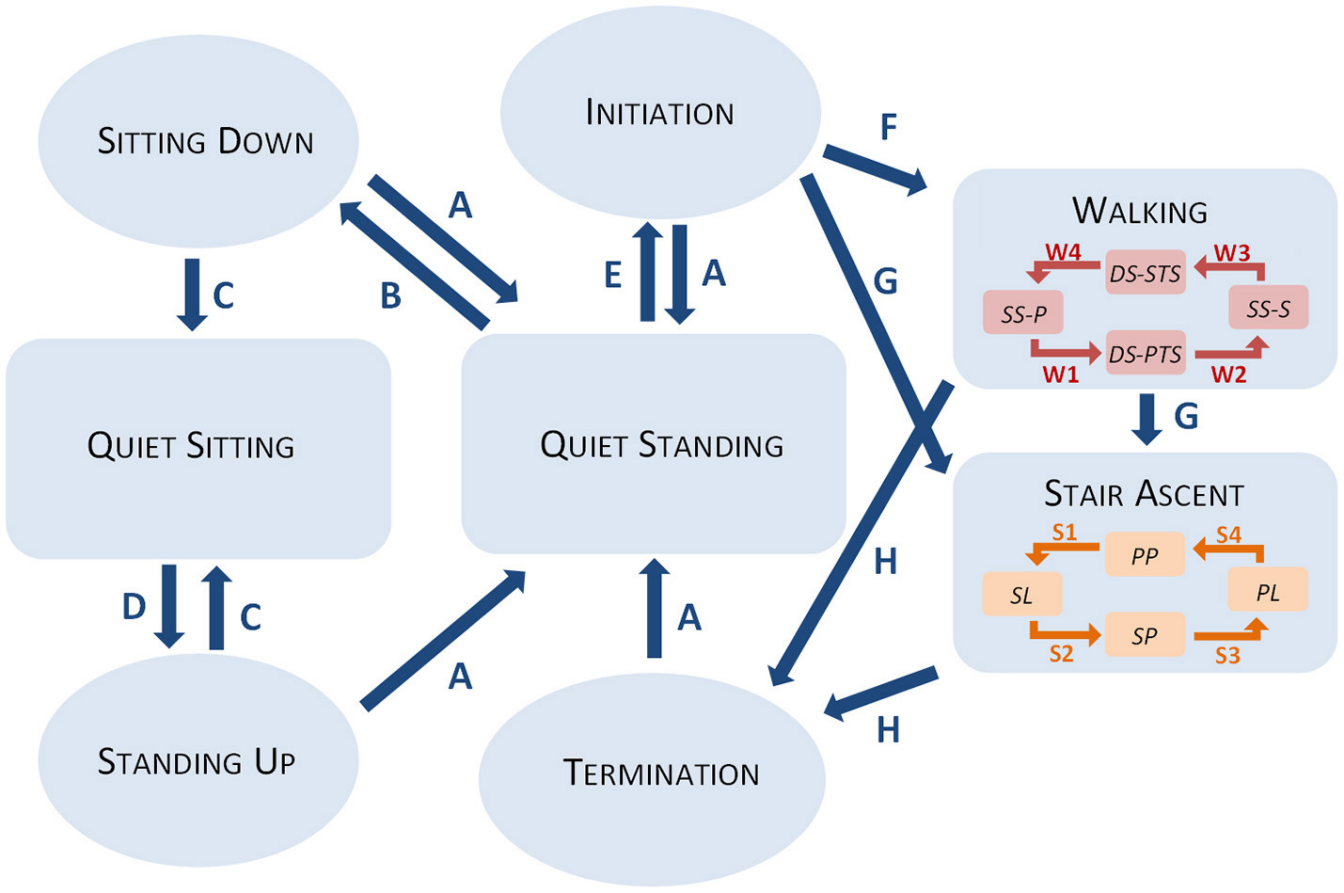
Conceptual block diagram of the recognized manouvres and allowed transitions between them. Each light blue block represents a state. Red and orange blocks within blue states represents subphases of the task. Arrows represents transitions between states labeled with letters. Letters are recalled in Table 1.

The controller distinguishes up to eight different maneuvres, among which four are steady state activities, while the other ones are transient actions. In particular, the controller recognizes (i) quiet standing, (ii) quiet sitting, (iii) step-by-step stair ascent, and (iv) walking, as steady state maneuvres, while the actions of (v) sitting down, (vi) standing up, (vii) initiating, and (viii) terminating locomotion are detected as transient states. Within each steady activity, the controller further recognizes the occurrence of subsequent subphases. Figure 2 shows a schematic picture of all the maneuvres distinguished by the controller and the associated allowed transitions. To be thorough, walking and stair ascent can only be entered upon recognition of specific subphases, i.e., any single stance phase in the first case, and step initiation with the sound limb, in the latter.

For either the detection of a new phase or activity, the transition rules consist of combinations of heuristic rules evaluating the exceedance of pre-set thresholds (Table 1). To account for inter-subject anthropometric differences, some thresholds—such as Ground Reaction Forces thresholds—were tailored on the specific subject in order to increase the robustness of recognition. Besides, additional rules guarantee safety in case of missed transitions' detections by timing out the current phase and triggering the next one or setting the prosthesis in the extended stiff configuration, preventing a state from lasting too long.

**Table 1:** Kinematic variables extracted from the WSA and used as input for the controller for implementing transition rules.

**ID input variables**	**Description**	
ω:	Angular velocity of the IMUs placed on right/left feet, shanks, and thighs [rad/s]	
θ:	Joint angles of right/left knee and hip [deg]	
GRF:	Ground reaction force [N]	
Sound_length, Prosthesis_length:	Hip-ankle distance at a given time normalized to the maximal leg length	
R_flat_, L_flat_:	Foot flat indicator [bool]	
**Activity and Subphases**	**Transition Rules**	
Quiet standing	A	|ω_R_thigh_| < 0,1 AND |ω_L_thigh_| < 0,1 AND |ω_R_shank_| < 0,1 AND |ω_L_shank_| < 0,1 AND |ω_R_foot_| < 0,1 AND |ω_L_foot_| < 0,1
Sitting Down	B	ω_R_thigh_ > 0,2 AND ω_L_thigh_ < −0,2 AND |ω_R_shank_| < 1,5 AND |ω_L_shank_| < 1,5 AND |ω_R_foot_| < 0,5 AND |ω_L_foot_| < 0,5 AND L_*flat*_ = = 1 AND R_flat_ = = 1
Quiet Sitting	C	|ω_R_thigh_| < 0,15 AND |ω_L_thigh_| < 0,15 AND |ω_R_shank_| < 0,15 AND |ω_L_shank_| < 0,15 AND |ω_R_foot_| < 0,15 AND |ω_L_foot_| < 0,15
Standing Up	D	ω_R_thigh_ < −0,5 AND ω_L_thigh_ > 0,5 AND |ω_R_shank_| < 1 AND |ω_L_shank_| < 1 AND |ω_R_foot_| < 0,5 AND |ω_L_foot_| < 0,5
Initiation	E	ω_R_foot_ < −0,5 OR ω_L_foot_ > 0,5
Walking	F	[(ω_L_foot_ > 0,7 AND ω_L_shank_ > 0,5) OR (ω_R_foot_ < −0,7 AND ω_R_shank_ < −0,5)] AND [NOT(L_flat_) OR NOT(R_flat_)]
DS-PTS Double Support—Prosthesis To Swing	W1	ω_sound_foot_ > 0 AND ω_sound_shank_ > 0 AND ω_sound_thigh_ > 0
SS-S Single Support—Sound	W2	ω_prosthesis_foot_ < 0 OR ω_prosthesis_shank_ < 0
DS-STS Double Support—Sound To Swing	W3	ω_sound_foot_ > 1 AND ω_prosthesis_shank_ < 0
SS-P Single Support—Prosthesis	W4	ω_sound_thigh_ < −1 AND ω_sound_foot_ < 1
Stair Ascent	G	GRF_prosthesis_ > GRF_thr_ AND GRF_sound_ < GRF_thr_ AND [(θ_Knee_sound_ > SA_K_Angle AND θ_Hip_sound_ > SA_H_Angle) OR (θ_Knee_sound_+ θ_Hip_sound_) > (SA_K_Angle+ SA_H_Angle)]
SL Sound Lifting	S1	GRF_prosthesis_ > GRF_thr_ AND (ω_sound_shank_ > 0, 5 OR ω_sound_foot_ > 0,5)
SP Sound Placement	S2	GRF_sound_>GRF_thr_ AND |ω_sound_foot_| < 0,1 AND ω_sound_thigh_ < 0
PL Prosthesis Lifting	S3	GRF_sound_ > GRF_thr_ AND GRF_prosthesis_ < GRF_thr_
PP Prosthesis Placement	S4	GRF_sound_ > GRF_thr_ AND Sound_length > SA_length
Termination	H	|ω_R_thigh_| < 0,1 AND |ω_L_thigh_| < 0,1 AND |ω_R_shank_| < 0,1 AND |ω_L_shank_| < 0,1 AND |ω_R_foot_| < 0,1 AND |ω_L_foot_| < 0,1

*Transition rules are labeled through the letters as the colored arrows in Figure 2*.

Once a specific maneuvre and phase are detected, the controller sets the commands that drive the actuation stage at the low level of control. During stair ascent and walking, the commands consist of cyclically locking/unlocking the Weight Acceptance and Energy Transfer mechanisms and setting the positions of the knee and ankle actuators, in order to make locomotion as efficient and natural as possible. Counter to this, during the stand-to-sit and sit-to-stand tasks, the control action is designed to assist the maneuvre by providing an extensor torque at knee level, thus resulting in additional push when standing up and in a supporting braking force while sitting down. A description of the control variables and their setpoints for each maneuvre are provided in Tables 2, 3. Additionally, a robustness loop is implemented for the control setpoints of the knee joint flexion/extension and Weight Acceptance blocking mechanism. The variation of their setpoint can be triggered not only by the transition from “Single Support—Sound” state to the “Double Support—Sound to Swing” state, but also by a threshold mechanism on the Centre of Pressure of the sound limb. Thereby, even if the transition due to segment angular speeds is not detected, the Centre of Pressure threshold enables knee extension and Weight Acceptance blocking to accomplish a safe initial foot contact with a rigid knee joint.

**Table 2:** Description of the control variables for the low-level controllers and their setpoints for the ATP actuated joints and mechanisms.

**Control variable**	**Unit**	**Setpoints**	**Description**	
Weight Acceptance Locking Mechanism	[mm]	Locked Unlocked	High knee joint stiffness Low knee joint stiffness	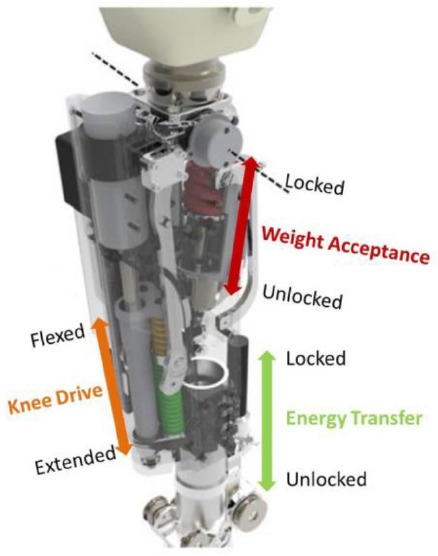
Energy Transfer Locking Mechanism	[mm]	Locked	Energy Transfer mechanism engaging knee and ankle joints	
		Unlocked	Energy Transfer mechanism not engaging knee and ankle joints	
Knee Drive Position	[mm]	Flexed	Knee joint in flexed configuration	
		Half-flexed	Knee joint in half-flexed configuration	
		Extended	Knee joint in extended configuration	
Ankle Motor Position	[deg]	Quiet Standing	Ankle angle during quiet standing	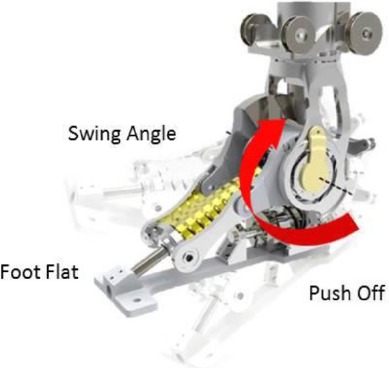
		Push off	Ankle angle during the push off phase	
		Swing Angle	Ankle angle during the swing phase	
		Flat Angle	Ankle angle during the foot flat phase	

**Table 3:** Description of the control setpoints commanded for each maneuvre and phase at the low-level controllers.

	**Control action**			
**Activity and subphases**	**Weigth acceptance locking mechanism**	**Energy transfer locking mechanism**	**Knee motor position**	**Ankle motor position**
Quiet standing	Locked	Unlocked	Extended	Quiet Standing
Sitting Down	Unlocked	Unlocked	Gradually flexed as f(θ_knee_, τ_knee_est__)	Quiet Standing
Quiet Sitting	Unlocked	Unlocked	Flexed	Quiet Standing
Standing Up	Gradually locked as f(θ_knee_)	Unlocked	Gradually extended as f(θ_knee_, τ_knee_est__)	Quiet Standing
Initiation	Locked	Unlocked	Extended	Quiet Standing
**WALKING**				
DS-PTS, Double Support—Prosthesis To Swing	Unlocked	Locked	Half-Flexed	Pushoff
SS-S, Single Support—Sound	Unlocked	Unlocked	Half-flexed	Swing Angle
DS-STS, Double Support—Sound To Swing	Locked (early stance)—Unlocked (late stance)	Unlocked (early stance)—Locked (late stance)	Half-flexed	Flat (early stance) -Pushoff (late stance)
SS-P, Single Support—Prosthesis	Locked (early stance)—Unlocked (late stance)	Unlocked (early stance)—Locked (late stance)	Half-flexed	Flat (early stance) -Pushoff (late stance)
**STAIR ASCENT**				
SL, Sound Lifting	Locked	Unlocked	Extended	Quiet Standing
SP, Sound Placement	Locked	Unlocked	Extended	Quiet Standing
PL, Prosthesis Lifting	Unlocked	Locked	Flexed	Swing Angle
PP, Prosthesis Placement	Gradually locked as f(θ_knee_)	Unlocked	Extended	Quiet Standing
Termination	Locked	Unlocked	Extended	Quiet Standing

From a hardware point of view, the controller runs on a reconfigurable input/output board sbRIO-9632 (National Instruments, Austin, Texas, US), endowed with a real-time processor and a Field Programmable Gate Array. This level runs at 100 Hz on the real-time processor and the code is developed with Labview Real-Time Module and Labview Statechart toolbox. Peripheral communication and trajectory tracking on the other hand, are managed by the FPGA at 1 kHz.

### Experimental protocol

Validation of the controller was performed through an experimental session involving four amputees wearing the CYBERLEGs ATP and the WSA while executing different locomotor tasks. The recruited subjects (age 63 ± 11; weight 61.7 ± 2.9; height 173.5 ± 4.8) had undergone amputation for different causes (three for traumatic reasons, while one was dysvascular) and for different durations, thus they exhibited considerably different levels of fitness and attitudes toward the prosthesis. Their mobility level in accordance with (Gailey et al., 2002) are reported among subject's characteristics. However, apart from the amputation, all the participants were in general good health. Their characteristics are reported in Table 4.

**Table 4:** Characteristics of the enrolled subjects.

	**Subject #1**	**Subject #2**	**Subject #3**	**Subject #4**
Age	67	72	65	48
Gender	M	M	M	M
Height	180	175	168	172
Weight	58	63	64	62
Year of amputation	11	2	4	26
Reason of amputation	Traumatic	Dysvascular	Traumatic	Traumatic
Mobility	K3	K1	K2	K3

Each subject wore the CYBERLEGs ATP together with the backpack housing the control unit of the whole system and the batteries for powering the actuators and the electronics. As for the WSA, the participants wore the shoes with the pressure-sensitive insoles and had seven IMUs placed on their body, more precisely on the trunk, the thighs, the shanks and the feet. The total setup time was no longer than 10 min for each participant.

The experimental protocol involved the repeated execution of four different maneuvres, covering all the activities detected by the algorithm:

- ground-level walking between parallel bars (GLW-PB), which consisted of repeated walks across a 6-m-long walkway. For safety reasons, ground-level walking was carried out on parallel bars equipped with handrails;
- treadmill walking (GLW-TM) at user's self-selected speed;
- stair ascent (SA), which involved the step-by-step climbing of a three-steps staircase;
- sit-to-stand and stand-to-sit (StS) cyclic movements.

**Figure 3:**
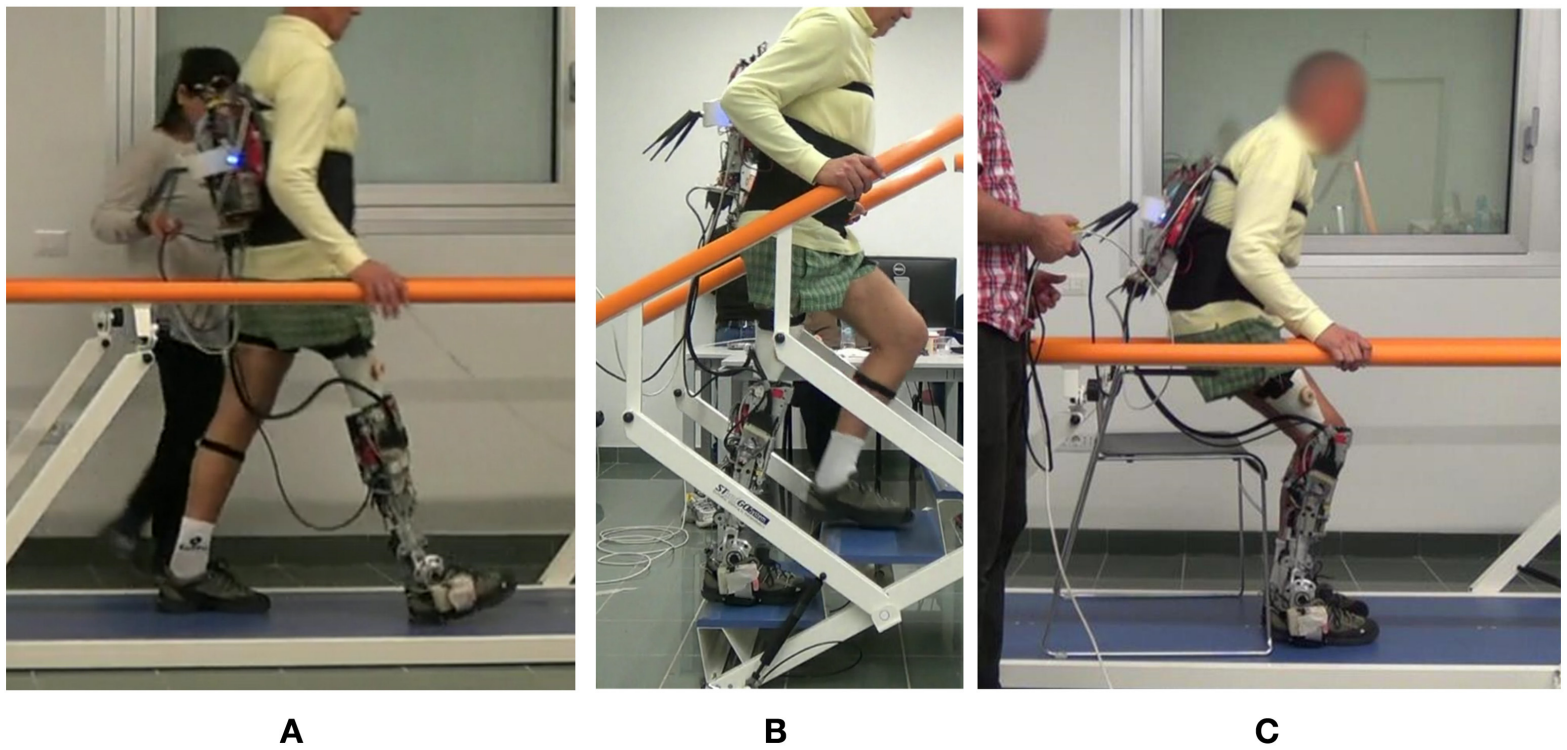
Overview of the experimental protocol with subjects performing: **(A)** ground-level walking between parallel bars, **(B)** stair ascent, and **(C)** sit-to-stand/stand-to-sit manouvres. The participant shown in this figure has provided written informed consent for the publication of this image.

Preliminary trials were performed to allow familiarization with the prosthesis and for fine tuning of the actuation setpoints according to each subject's anthropometry and to gain familiarity and confidence with the prosthesis. The recording session subsequently took place and required the following minimum numbers of accomplished repetitions for each maneuvre, in order to provide a sufficient amount of data for significant statistical analysis: 10 walks for each parallel bars trial; at least 2 min of treadmill walking; 15 steps (three-steps staircase × 5 times) for stair ascent; 10 cycles of sit-to-stand and stand-to-sit maneuvres. An overview of one of the enrolled participants performing different activities is reported in Figure 3.

This study was reviewed and approved by the Ethical Committee of the Fondazione Don Gnocchi (Florence, Italy), where the experiments were conducted. All subjects gave written informed consent in accordance with the Declaration of Helsinki.

### Data analysis

Recorded data were analyzed in Matlab. Each trial was first segmented into single repetitions of each maneuvre. Then, every single maneuvre was further divided into its subphases.

Segmented data were analyzed to investigate accuracy of the intention detection algorithm at two levels: first, (i) accuracy in maneuvre detection was computed in terms of success rate, i.e., the ratio of the number of successfully recognized instances of each activity to the number of total performed repetitions of the same activity. Then, (ii) accuracy in the recognition of the subphases of each maneuvre was evaluated in an analogous way, by computing the success rate of each subphase detection. At this level, only correctly identified repetitions were considered. To assess whether a certain maneuvre or phase had been correctly recognized, a reference signal indicating the actual performed task was computed offline from the data recorded by the WSA. This signal was then compared with the output of the intention detection controller in order to evaluate its performances in terms of success rates of detection.

## Results

### Ground-level walking on treadmill

**Figure 4:**
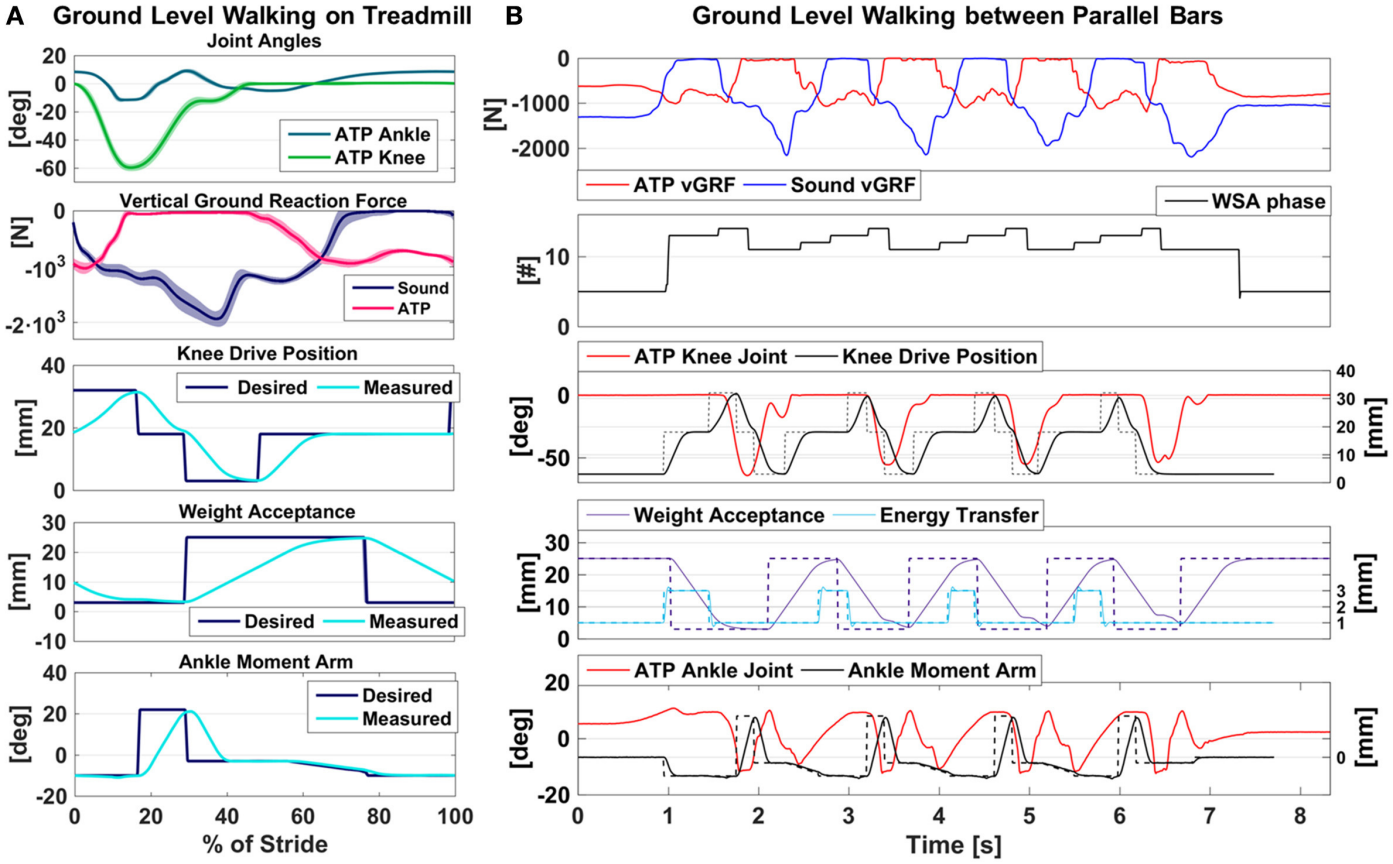
Overview of the ATP kinematics during GLW tasks. **(A)** GLW-TM: steady-state gait variables from a representative subjects were segmented with the sound limb heel strike and averaged across strides. Averaged profiles as a percentage of the gait cycle are reported contoured by the standard deviation. **(B)** GLW-PB: Time series of the gait variables from a representative trial of a representative subject while walking between parallel bars.

Treadmill walking trials aimed at testing the efficacy of the WBAC algorithm and studying the extent of subjects' cooperation with the active device, during a steadily cyclic task. Figure 4 displays the averaged kinematic profiles and the main actuation trajectories of the prosthesis for a representative subject, landmarking the heel strike of the sound limb as the beginning of the stride. Only steady-state strides were considered. Strides during initial acceleration and final deceleration of the treadmill are excluded.

Besides showing repeatability of the task as proved by moderate standard deviations, the reported graphs confirm the expected behavior of the actuation: taking as reference the heel strike of the sound limb, the prosthetic knee is kept flexed in this phase, being the ATP in its late stance. At the same time, the ankle—which is in the push-off phase—is slightly plantarflexed. Once the prosthesis enters the swing phase, the knee is commanded to the half-flexed configuration, while the ankle is dorsiflexed to provide sufficient ground clearance. Thus far, the weight acceptance mechanism is maintained unlocked, since the amputee shifted the weight to the sound limb. Then, during the prosthesis swing phase, preparation for the forthcoming heel strike occurs: the ankle is brought in the quiet standing position, i.e., about parallel to the ground, the knee is extended and the weight acceptance is locked.

Concerning the intention detection capability of the algorithm, overall accuracy in recognizing the different gait phases is displayed in the upper section of Table 5. It is worth noting that Subject #2, as the K1 amputee, was able to perform just 13 strides on the treadmill before asking to stop for exhaustion. Nevertheless, the accuracy rate was 100% also in his performance. Recognition of the initiation and termination states was also considered, since the initial acceleration and final deceleration of the treadmill are included in the task. With regards to this, the algorithm resulted robust to such transient phases, successfully recognizing 100% of movement initiations and terminations.

**Table 5:** Success rate of the GLW-TM and GLW-PB tasks.

	**SS-S, Single Support-Sound**		**DS-STS, Double Support-Sound To Swing**		**SS-P, Single Support-Prosthesis**		**DS-PTS, Double Support-Prosthesis To Swing**		**Initiation**		**Termination**	
**GLW-TM**												
#1	337	337	335	335	337	337	336	336	4	4	4	4
#2	14	14	13	13	14	14	14	14	1	1	1	1
#3	65	65	64	64	65	65	65	65	1	1	1	1
#4	567	567	564	564	567	567	567	567	3	3	3	3
All subjects	983	983	976	976	983	983	982	982	9	9	9	9
SR [%]	100.0		100.0		100.0		100.0		100.0		100.0	
**GLW-PB**												
#1	83	83	76	76	83	83	74	74	20	20	19	20
#2	73	78	63	67	79	82	77	79	17	17	16	17
#3	171	174	138	142	174	177	173	175	34	34	34	34
#4	141	152	122	134	133	140	132	138	27	31	31	31
All subjects	468	487	399	419	469	482	456	466	98	102		102
SR [%]	96.1		95.2		97.3		97.9		96.1		98.0	

*For each subject, the number of occurrence of the subphases is reported together with the number of correct classification*.

### Ground-level walking on parallel bars

From a kinematic point of view, parallel bars trials are similar to treadmill ones. However, walking on parallel bars holds an increased level of complexity, for it requires greater coordination from the subject to walk over the ground and to start, slow down and stop the movement. Figure 4 shows a representative trial for Subject #1. Essentially, prosthesis actuation is achieved for GLW-PB as for the GLW-TM maneuvre, with even the setpoints being unchanged. Yet, initiation and termination require detailed considerations. Initiation is recognized as at least one angular speed at the foot exceeding a certain threshold. Then, as soon as level-ground walking is recognized as the ongoing maneuvre, the prosthesis is actuated depending on whether the stance limb is the sound one or the prosthetic one. Prior to this moment—and, more in general, to the recognition of an activity—no action is applied on the prosthesis. Termination and subsequent quiet standing recognition occur when all the monitored angular speeds of body segments approach zero. At this moment, the prosthesis is brought back in the quiet standing configuration, i.e., with the knee extended and stiff knee and ankle parallel to the ground.

The more challenging nature of the GLW-PB maneuvre is found also in the decoding of the motor intentions of the person, since the less regular execution of the walking task demands for an intrinsically robust algorithm to maintain high accuracy levels. Besides efficacy in the detection of cyclic gait phases, during GLW-PB maneuvre it is also assessed the ability of the algorithm in recognizing initiation and termination at the beginning and at the end of each passage.

The lower portion of Table 5 summarizes the results of success rate computation. Although not being visible from the table, misdetections had different causes depending on the subject. In particular, for Subjects #2 and #3, the algorithm was deceived by the low walking speed, which resulted in detection of termination despite the ongoing task. Differently, for Subject #4 and #3 most errors occurred during the first and the last steps, thus being related to gait onset and conclusion. Explanation for such behavior lays in how transition rules for the considered activities are conceived i.e., based on evaluation of body segments' angular speeds, and in the one-way cyclic nature of the ID algorithm.

To make a thorough analysis of the accuracy of the controller, an evaluation of the undetected transitions at the initiation and termination of each locomotion activity was also performed. To this extent, two confusion matrixes were computed, to respectively account for missed transitions occurring (i) at the initiation (Table 6A) and (ii) at the termination of the movement (Table 6B). Considering ground level walking, in 3.6% of cases, the incipient movement was not recognized, while the intention to stop was not detected with 1.8% error rate. Contrarily, only 0.1% error rate for termination was detected despite not being intended by the subject, a result in accordance with the observations previously made for Subjects #2 and #3.

**Table 6:** Confusion matrix of the Intended vs. Detected transitions occurring at the **(A)** onset of each activity and **(B)** end of the steady activities.

**A**		**Detected Transition**				**B**		**Detected Transition**			
		**QS > QS**	**QS > StS**	**QS > SA**	**QS > GLW**			**GLW > GLW**	**GLW > QS**	**SA > SA**	**SA > QS**
Intended	QS > QS	100%	0%	0%	0%	Intended	GLW > GLW	99.9%	0.1%	x	x
Transition	QS > StS	2.5%	97.5%	0%	0%	Transition	GLW > QS	1.8%	98.2%	x	x
	QS > SA	0%	0%	91.7%	8.3%		SA > SA	x	x	100%	0%
	QS > GLW	3.6%	0%	0%	96.4%		SA > QS	x	x	0%	100%

*Percentage values were obtained for all the subjects during all the trials. GLW, Ground Level Walking; QS, Quiet Standing; SA, Stair Ascent*.

### Stair ascent

**Figure 5:**
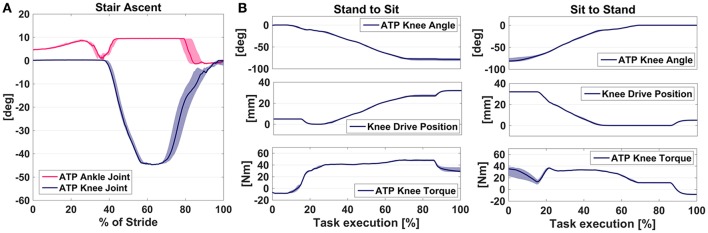
Overview of the ATP kinematics during: **(A)** SA tasks: gait variables from a representative subjects were segmented with the sound limb foot contact and averaged across strides on the stairs. Averaged profiles as a percentage of the step cycle are reported contoured by the standard deviation; **(B)** StS: gait variables from a representative subjects were segmented on the task execution and averaged across trials. Averaged profiles as a percentage of the task execution are reported contoured by the standard deviation.

SA was performed one step at a time, lifting and placing the sound limb first, and then completing the same step with the prosthetic one. For this maneuvre, the ATP was actuated in order to effortlessly provide the necessary clearance to climb the step avoiding compensatory movements at pelvis level: basically, as soon as the prosthetic limb is raised, the knee is flexed and the ankle is dorsiflexed; then, before foot landing, the first one is gradually extended while the latter is brought back to the flat position, as can be observed from Figure 5. As for the phases of prosthesis support, Figure 5 shows the knee kept extended (and stiff) to provide support and the ankle being in the flat configuration.

Accuracy here was investigated as the ability of the algorithm (i) to detect SA activity, and then (ii) to correctly identify its subphases. Inspection of Table 7 shows reliable phase detection, only spoiled by Subject #1 attempting to climb the steps quickly. As for the activity recognition, few missed detections are observed for Subjects #2 and #4. In these cases, as shown in Table 6A, detection of Stair Ascent did not occur against a false recognition of Ground Level Walking.

**Table 7:** Success rate of the SA task.

**SA**	**SA, Activity**		**SL, Sound lifting**		**SP, Sound placement**		**PL, Prosthesis Lifting**		**PP, Prosthesis placement**	
#1	24	24	22	24	22	24	22	24	22	24
#2	10	12	10	10	10	10	10	10	10	10
#3	9	9	9	9	9	9	9	9	9	9
#4	23	27	23	23	23	23	23	23	23	23
All subjects	66	72	64	66	64	66	64	66	64	66
SR [%]	91.7		97.0		97.0		97.0		97.0	

*For each subject, the number of occurrence of the subphases is reported together with the number of correct classification*.

### Stand-to-sit and sit-to-stand

StS is the maneuvre involving active power delivery as extensor torque at the knee joint. Bottom plots of Figure 5 depict how the assistive torque is supplied at knee level during task accomplishment. In both stand-to-sit and sit-to-stand, the assistive torque is extensor—respectively, acting with damper effect and as a pushing force, as explained in the previous section—while the resulting trend of its amplitude is opposite for the two tasks, i.e., increasing torque magnitude when sitting down and decreasing it while standing up.

Results of the intention detection are displayed in Table 8. It is worth noting the effect of the sequential nature of the algorithm on the states recognition: the algorithm is initialized in quiet standing and any phase can be detected unless the previous one has, implying inevitably lower or equal success rates for subsequent states recognition. In case of missed detection of the intention to sit down, which occurred with an error rate of 2.5% (Table 6), the prosthesis remained in the Quiet Standing configuration.

**Table 8:** Success rate of the StS task.

**StS**	**Sitting Down**		**Quiet Sitting**		**Stading Up**		**Quiet Standing**	
#1	29	29	27	29	25	29	25	29
#2	12	12	12	12	12	12	12	12
#3	22	22	22	22	22	22	22	22
#4	14	14	14	14	14	14	14	14
All subjects	77	77	75	77	73	77	73	77
SR [%]	100.0		97.4		94.8		94.8	

*For each subject, the number of occurrence of the subphases is reported together with the number of correct classification*.

## Discussions and conclusions

In this paper, a novel WBAC based on a distributed WSA for controlling an ATP was presented. The WBAC recognizes different ambulatory activities based on a simple set of heuristic rules providing real-time subject-independent classification using non-invasive mechanical sensing. The intent recognition is the basis for commanding desired setpoints for the low-level controllers of the actuated mechanisms with the ultimate goal of providing support to the transfemoral amputees during ground-level walking, stair ascent and sit-to-stand maneuvres. As the main purpose of this study is the assessment of the WBAC controller employed for the ATP, discussion of the results will focus on the accuracy of the motor recognition rather than the effectiveness of the applied control action. However, since the strategy adopted to drive the prosthesis has an influence on the kinematics of the user, the effects of the actuation will be considered for controller developments.

In the framework of intent recognition algorithms for powered prosthesis, the main novelty with respect to most of the state-of-art contribution is the fact that the WBAC avoids the use of neuro-related signals. Use-related issues of EMG degrade in most of the cases the robustness of the controllers. This has been recently confirmed, at least in simulation environment, in the work presented in Spanias et al. (2016) in which an adaptive controller was programmed to disregard neural interfaces for performing intent recognition method in an architecture based on neuromechanical sensing, when the sEMG signals are corrupted due to prolonged use. Performance was kept unvaried when the recognition is based only on the mechanical sensing components and not considering the sEMG contribution when drifts are detected. Besides, recognition success rate of our algorithm was not lower than 94.8% in performing locomotion-related activities which is comparable with many studies in which neuromechanical interfaces were considered (Jin et al., 2006; Huang et al., 2009, 2011; Ceseracciu et al., 2010; Tkach and Hargrove, 2013).

The experimental session with amputees provided relevant feedback on the general working of the tested controller and the extent of its acceptability for the end-users. Results demonstrated overall correct functioning of the algorithm, both in terms of repeatability of the resulting kinematic profiles and accuracy of the intention detection. At the same time, these tests highlighted the major criticalities of the current controller and allowed a deeper understanding of their causes; such main limitations will be hereafter discussed and addressed in future revisions of the algorithm.

One of the main outcomes is the accurate classification of the subphases of steady dynamic maneuvres such as treadmill walking and stair ascent. Remarkably, the algorithm achieved full success rate for subphases recognition during treadmill walking and 97% for stair ascent thanks to robust one-way sequential architecture of the recognition path. In fact, as soon as the intended maneuvre is detected along with the correct subphase, the following phases are recognized in a sequential way, resulting in the establishment of an effective loop: successful classification of the current phase triggers a control action promoting the natural progression of the task cycle, so that the user is smoothly guided to the next phase. Sources of misclassification can be related to unexpected events as obstacle avoidance.

Treadmill walking was not considered in the validation activities of the former controller. The proposed controller is capable of providing 100% success rate while steadily walking on treadmill even in case of very slow speed as for subject #2 walking at 1.4 km/h. This is a remarkable aspect of the new version of the algorithm allowing for the feasibility of rehabilitation of non-ambulatory K1 subjects in early rehabilitation phases. The same result was achieved during steady locomotion only in Maqbool et al. (2016) for ground-level walking using foot-placed gyroscopes. As it regards the performance benchmark against other methods based only on mechanical sensing found in the state of the art, the performance of our algorithm resulted in similar accuracy rate with respect to the results achieved in Chen et al. (2015) and Yuan et al. (2015). Classification accuracy reached 98.4 and 98.7%, respectively; nevertheless, in both study, the validation involved only transtibial amputees, in particular only one subject for the first and three subjects for the latter study.

However, in the case of GLW-PB—thus when applying the aforementioned strategy overground—speed becomes a critical parameter for proper functioning of the algorithm. In fact, in some cases too low angular velocities of the body segments affected success of recognition by triggering termination when the subject was not actually willing to stop interrupting the movement during the stance phase until the algorithm recognized the ongoing maneuvre once again. This condition only occurred for the two weakest subjects, namely Subjects #2 (K1 mobility) and #3 (K2 mobility). Nevertheless, it did not result in any unsafe condition, since every time termination is detected, the prosthesis is driven back to the quiet standing configuration in which the command action for the ATP resembles the mid stance phase of steady walking.

The other main source of incorrect detections displayed for overground walking regarded erroneous classifications of the first phase detection following gait initiation and of the last phase detected before gait termination. For the first case, the detected phase is shifted with respect to the actual one, and, given the sequential structure of the algorithm, it might take a stride period to enter again in the actual one and reach the steady state. This is particularly disruptive in case initiation is detected for the right prosthetic limb while it is still in the monolateral support phase. The result is the incurrence in a false swing with a consequent unblock of the knee. By the way, such erroneous behavior was observed only for one subject, so the extent of significance of this error would need further investigation.

As for task conclusion, if the user is stopping slowly, the algorithm may not detect any further transitions until all the speeds fall under the threshold for termination recognition, thus not updating the control action even if the user gait phase is progressing. Nevertheless, incorrect classifications at both gait onset and conclusion did not affect more than one stride each, nor did the participants report any perception of troublesome conditions. Besides, considered that the subjects did not receive an actual training, it is not to be excluded that with prolonged use of the device and gaining confidence in the prosthesis, such behavior would be anyway doomed to diminish.

Since GLW-PB had already been tested for the previous controller, it is also possible a rough comparison with the results of the previous controller (Ambrozic et al., 2014). Indeed, Subjects #1 and #3 of this experiment participated also in the activities reported in Ambrozic et al. (2014) as Subject #1 and Subject #3, respectively. The accuracy rate for Subject #1 in Ambrozic et al. (2014) was not higher than 96.2% in all the subphases. If we analyze the success rate for GLW-PB only for Subject #1 we obtain a success rate of 100% for all the subphases except for the gait termination with 95.0%, still higher than the one obtained in Ambrozic et al. (2014). For Subject #3, the success rate is comparable for most of the subphases. Importantly, in the “Double Support—Sound to Swing” state the accuracy rate has improved from 88.9 to 97.2%. We obtained also 100% success rate in Gait Initiation and Termination against 96.0 and 64.0%, respectively, in the previous version of the controller (Ambrozic et al., 2014). Furthermore, the averaged success rate for gait initiation and termination in Ambrozic et al. (2014) was 85.2 and 64.8%, respectively. The new controller demonstrated higher capabilities to correctly identify those modes with a success rate of 96.1 and 98.0%, respectively. With regards to safety, an increased success rate is a remarkable improvement of the proposed WBAC with respect to its previous version.

Concerning the results obtained for the remaining maneuvres, generally accurate detection was reported, with only minor errors not related to systematic errors intrinsic to the algorithm, but rather to suboptimal threshold settings, as in the cases of missed SA maneuvre detection for Subject #4 or missed StS activities detection for Subject #1.

All of these issues considered, the algorithm achieved satisfactory outcomes in terms of accuracy, especially considering that the users were not trained to use the prosthesis and that transition rules are mostly subject-independent. In this perspective, results confirmed that the developed algorithm reached an acceptable tradeoff between accuracy of detection and intuitiveness of use, which was the primary objective of this work.

Most importantly, even in case of a fault in the detection, either due to a missed transition or to a wrong activity detection, the algorithm never resulted unsafe for the user, which is another mandatory requirement for a prosthesis controller: the architecture of the recognized states and allowed transitions is attentively conceived to (i) separate dynamic from static tasks and (ii) implement recognition in a hierarchical way, implicating that it is not possible to detect any phase of a dynamic maneuvre unless initiation of a dynamic movement has been previously recognized, as well as it is not possible to detect a certain phase of a cycle (such as StS cycle, gait cycle, step cycle) without detection of the previous one. In this way, the user is prevented from completely inappropriate control actions that may result in disruptive conditions.

Moreover, despite not yet reaching optimal levels of accuracy in recognizing the user's intentions, the architecture of the controller makes it extremely improbable to detect an ongoing task when not actually being performed. Such eventuality may trigger unbalancing control actions and arguably, in terms of reliability and thus acceptability for end-users, it is preferable a lower sensibility of the controller in favor of a higher recognition robustness.

Importantly, one of the current limitations of this work is the lack of robust strategies to cope with abrupt speed variations. Even if robustness loops on measurements that are invariant with respect to the gait cycles are adopted, therefore improving speed-independency, these loops are suitable to manage slow speed variations. As well, locomotion-related activities such as slope walking or approaching of uncertain terrains have not been object of this study.

Future works will be focused on the improvement of the reliability of the WBAC by means of a transitioning rules design revision based on the feedback collected from these experimental activities. Furthermore, speed-independency strategies, slope walking and seamless transitions between states will be designed. Reduction of the number of sensitive elements of the wearable interface will be explored.

## Author contributions

AP carried out the experimental activities and data analysis, participated in the design of the study and drafted the manuscript. EM and SC significantly contributed to the data analysis and drafted the manuscript. JG, LF, and GP participated in the design of the study and carried out the experimental activities. RM participated in the design and coordination of the study. RK and DL conceived the study and participated in its design. MM and NV conceived the study, participated in the design and coordination of the study. All authors approved the submitted version of the manuscript.

### Conflict of interest statement

The authors declare that the research was conducted in the absence of any commercial or financial relationships that could be construed as a potential conflict of interest.

## Abbreviations

ATP: Active Transfemoral Prosthesis
DS-PTS: Double Support—Prosthesis to Swing
DS-STS: Double Support—Sound to Swing
sEMG: Surface Electromyography
GLW-PB: Ground-level walking on parallel bars
GLW-TM: Ground-level walking on treadmill
IMU: Inertial Measurement Unit
LDA: Linear discriminant analysis
PL: Prosthesis Lifting
PP: Prosthesis Placement
SA: Stair Ascent
SL: Sound Lifting
SP: Sound Placement
SS-P: Single Support—Prosthesis
SS-S: Single Support–Sound
StS: Sit-to-Stand/Stand-to-Sit
WBAC: Whole Body Awareness Controller
WSA: Wearable Sensory Apparatus.
